# Predicting the risk of hospital readmissions using a machine learning approach: a case study on patients undergoing skin procedures

**DOI:** 10.3389/frai.2023.1213378

**Published:** 2024-01-05

**Authors:** Jigar Adhiya, Behrad Barghi, Nasibeh Azadeh-Fard

**Affiliations:** Industrial and Systems Engineering Department, Kate Gleason College of Engineering, Rochester Institute of Technology (RIT), Rochester, NY, United States

**Keywords:** healthcare, hospital readmissions, machine learning, skin procedures, health outcome prediction, risk prediction

## Abstract

**Introduction:**

Even with modern advancements in medical care, one of the persistent challenges hospitals face is the frequent readmission of patients. These recurrent admissions not only escalate healthcare expenses but also amplify mental and emotional strain on patients.

**Methods:**

This research delved into two primary areas: unraveling the pivotal factors causing the readmissions, specifically targeting patients who underwent dermatological treatments, and determining the optimal machine learning algorithms that can foresee potential readmissions with higher accuracy.

**Results:**

Among the multitude of algorithms tested, including logistic regression (LR), support vector machine (SVM), random forest (RF), Naïve Bayesian (NB), artificial neural network (ANN), xgboost (XG), and k-nearest neighbor (KNN), it was noted that two models—XG and RF—stood out in their prediction prowess. A closer inspection of the data brought to light certain patterns. For instance, male patients and those between the ages of 21 and 40 had a propensity to be readmitted more frequently. Moreover, the months of March and April witnessed a spike in these readmissions, with ~6% of the patients returning within just a month after their first admission.

**Discussion:**

Upon further analysis, specific determinants such as the patient's age and the specific hospital where they were treated emerged as key indicators influencing the likelihood of their readmission.

## 1 Introduction

Hospital readmission is defined as an act of readmitting the patient to a healthcare facility after being discharged for prior admission (Mayo Clinic, 2020). The healthcare industry in the United States has faced issues caused by readmissions. They impose side effects on the healthcare costs and quality of care provided by the facilities (Basu et al., 2016). Readmissions to healthcare facilities are often perceived as preventable and expensive events (Weissman et al., 1999; Jiang et al., 2003; Jayasree and Friedman, 2004; McIlvennan et al., 2015). Sometimes, preventable readmissions are considered an indicator of poor quality of service provided by healthcare facilities (Goldfield et al., 2008; Dshs.Texas.Gov, 2020). Readmission results in the re-utilization of necessary resources by individual patients, making those resources unavailable to other patients. Readmission has been classified into planned and unplanned readmissions based on the discharge diagnosis (Kossovsky et al., 1999a). As per the Centers for Medicare and Medicaid Services (CMS), planned readmissions are predetermined at the time of last discharge (Archer and Marmor, 2012).^1^ On the other hand, unplanned readmissions occur without any prior knowledge and have at least one of the primary discharge diagnoses. This type of readmission is generally considered the primary indicator of the quality of care provided by the healthcare systems (Kossovsky et al., 1999b). One of the most significant types of readmissions involves cases where a patient is readmitted for a reason clinically related to the prior hospitalization within a specified time interval (Zhang et al., 2019).

A better understanding of the importance of reducing readmission can be sought by considering an example of the current scenario when the world is facing a pandemic of novel COVID-19. It has become crystal clear that we may not have enough medical resources to care for all the patients in need of care (Parker et al., 2020). Additionally, readmitting patients during such a scenario would exponentially increase their risk of being infected, which could further cause fear and mental trauma in patients. Hence, to prepare our healthcare for the future, it becomes essential to monitor the performance of healthcare providers and reduce the readmission rates. The Hospital Readmission Reduction Program (HRRP) was applied by the Department of Human Health and Services (HSS) to reduce the payments to the Inpatient Prospective Payment System (IPPS) for hospitals with excess readmissions beginning from the 2013 fiscal year (FY). Starting from the FY 2019, the 21st Century Cures Act necessitated the CMS to utilize the HRRP for reducing the readmissions for health conditions. In the end, six health conditions and procedures were identified (CMS, 2020).

In the study, patient readmissions after skin treatments are examined, given the significance of skin as an essential health indicator. Given the vital role of the skin, ensuring its best health is paramount to guarantee the overall wellbeing of a patient (Becker's Hospital Review, 2020). Some skin procedures include removing lesions, rearranging skin tissue, and skin graft procedures. The statistics in 2018 showed that there were 83,996 new melanomas of the skin in the United States and 8,199 people died.^2^ In addition, 4,237 cancer cases were reported in the NY state. Although skin treatment does not lie among the six crucial conditions CMS considers for HRRP; the literature shows that readmission post-skin treatments have not been studied well (Arnold et al., 2018; Zhang et al., 2019). Moreover, the applied data show that the highest number of readmissions occurred after skin treatments. Therefore, the focus of this study is to study the risk of hospital readmissions after skin treatments.

Moreover, the readmission timeframe highly studied by researchers is 30 days since the HRRP program penalizes hospitals for having higher than the expected 30-day readmission rates (CMS, 2020). However, the literature shows that readmissions within shorter timeframes, e.g., 7 days, are a better indicator of the quality of care (Chin et al., 2016). Additionally, a study also considered larger timeframes, such as 45 days, 90 days, and 1 year, to identify the best time interval that could account for unplanned readmissions among patients undergoing hepatectomy (Brudvik et al., 2015). Previous literature has analyzed and predicted readmission rates at specific time intervals and variables (Chin et al., 2016; CMS, 2020). However, a comprehensive study focusing on all the factors such as place of service, claim type, and line of business (LOB) to determine the readmission rates for skin condition patients has not yet been performed. Additionally, previous research has used traditional descriptive statistical techniques to analyze readmissions among patients undergoing skin treatments; no study has yet been conducted to predict the risk of readmission in these patients using different machine learning models. Therefore, the main objective of this study is to predict the risk of hospital readmissions that occurs at 30-day time interval post-skin treatment procedures. Seven machine learning models with seven features, such as the patient's age, gender, claim type, line of business (LOB), month of admission, hospital key, and Health Cost Grouper (HCG) subcategory, were applied to predict and analyze readmissions.

The remainder of the study flows as follows: First, related literature is presented in Section 2. Then, in Section 3, the methodology used in this study is provided. The results of the experiments are shown in Section 4. Finally, the discussion of the results and conclusions are presented in Section 5.

## 2 Literature review

Studies have been carried out in the past that tried to identify the relationship between health conditions and readmission rates for various disease diagnoses (Donzé et al., 2013). Such studies aimed to determine if the readmissions differ for multiple health conditions and whether the readmissions occurring for a particular health condition are higher than the others. These types of studies would help healthcare providers and administration to narrow their search and target the health conditions, resulting in a large number of readmissions. This could also aid in reducing the time required to come up with readmission reduction techniques for such high readmission rates. Furthermore, research has been carried out to analyze the factors causing readmissions for patients with the health conditions included in HRRP (Philbin et al., 2001; Rodríguez-Artalejo et al., 2006; Ali and Gibbons, 2017). Most of these studies came up with outcomes that suggested a significant relationship between patient demographics and the corresponding readmission rates. A study (Ali and Gibbons, 2017) showed that hospital readmission following a hip fracture could be associated with the patient's age and comorbidities. Another study regarding readmissions among heart failure patients (Rodríguez-Artalejo et al., 2006) indicated that socially active patients had lower readmission rates than socially inactive patients. Furthermore, one study (Philbin et al., 2001) found that the lower income of a patient is a positive predictor of readmission rates.

In addition to patient-related factors, the physician was also considered one of the predictors of readmission [Institute of Medicine (US) Division of Health Sciences Policy, 1983; Rauch et al., 2018]. The differences in the readmission rates after general surgery procedures based on physicians' gender were examined in another study (Wallis et al., 2022). This study discussed that there might exist differences in surgeries based on the sex of the physicians who performed them. Some of the critical differences in carrying out medical procedures by male and female physicians include adherence to clinical guidelines, frequency of preventive care, means of examination, and psychological counseling. Female physicians were better than their male counterparts in all of these measures. This study also revealed that the patients operated by female physicians had lower 30-day readmission rates. In addition, another study conducted among 1.3 million patients revealed that female patients treated by male surgeons had 15% greater odds of worse outcomes and 32% more likely to die than female patients treated by female surgeons (Wallis et al., 2022).

In addition to the personnel involved in hospitalization, the quality of care also depends on factors related to healthcare settings (Mosadeghrad, 2014). One of the essential performance metrics for healthcare settings is the hospital length of stay (LOS) (Mohajon, 2020). LOS was defined as the total duration of time in terms of days that a patient spends in the hospital during a single admission (Thefreedictionary.Com, 2020). Another study (Baek et al., 2018) suggested that patients receiving a poor quality of care have a higher LOS. This study also suggested that decreased LOS is connected to a reduced risk of hospital-acquired infections and medication side effects. A number of studies have been performed previously to develop a relationship between readmission rates and LOS (Altman et al., 1973; Lin et al., 2006; Kaboli et al., 2012; Vorhies et al., 2012). Some of these studies found an inverse relationship between patients' LOS and readmission rates, meaning that the admits who have a lower length of stay have higher readmission rates (Lin et al., 2006; Ofir and Padman, 2018). Other studies concluded that there is no significant dependence of readmission rates on the length of hospital stay (Altman et al., 1973; Vorhies et al., 2012).

Additionally, research has been conducted to check if factors such as insurance provider, hospital type, international classification of disease (ICD) codes, patient's race, and marital status affect hospital readmission rates (Kassin et al., 2012; Dailey et al., 2013). The authors of another study (Kassin et al., 2012) aimed to identify the risk factors for 30-day readmission among general surgery patients. This study proposed that the most common reasons for readmissions were gastrointestinal problems/complications (27.6%), surgical infection (22.1%), and failure to thrive/malnutrition (10.4%). Multivariate analysis demonstrated that postoperative complications were a primary reason driving readmissions in surgical patients. Analyzing the risk factors of the readmissions in post-orthopedic surgeries showed that admission to the intensive care unit gives the highest odds ratio of 2.365 for 30-day readmissions (Dailey et al., 2013). This study also indicated that patients' race, marital status, and Medicaid insurance status could reflect the patients' socioeconomic standing, further impacting their probabilities of readmission.

Another crucial variable considered for understanding the characteristics of the readmissions is the time interval in which the readmission occurs (Dorajoo et al., 2017; Mahle et al., 2019; Weiss et al., 2020). One of the recent studies for readmissions post-heart transplant focused on readmissions occurring within 30 days and 1 year after the index discharge (Mahle et al., 2019). Their results showed that the highest risk of readmissions lay within the first 30 days. An additional recent study performed to analyze the causes of heart surgery readmissions concentrated on two different timeframes (Weiss et al., 2020). One of the timeframes concentrated on readmissions within 30 days, whereas the other focused on readmissions occurring between the 31st and 180th days after the previous discharge. The findings of this study suggested that the leading cause of readmissions differed for these timeframes. Pleural effusion was the primary cause of readmissions within 30 days, and infection was the major cause for readmissions occurring between the 31st and 180th days. Another study (Dorajoo et al., 2017) concentrated on readmissions occurring within 15 days as a risk of early readmissions. Through this study, a model was developed that suggested that premature discharge could be one of the reasons for early readmissions. Five machine learning methods were compared for 30 days of readmission risk prediction (Ofir and Padman, 2018). This study showed that the boosted decision tree model was superior to the other models regarding accuracy and AUC measurements. The cost-effectiveness of intervention strategies was applied by a study (Lee et al., 2018) to reduce total joint replacement (TJR) readmission using a developed boosting machine learning framework within 90 days after discharge. In one study (Yu and Xie, 2020), a joint-ensemble algorithm, a data-driven approach, was designed to overcome the challenges of readmission prediction using electronic health records, utilizing a nationwide healthcare dataset.

Despite numerous studies on analyzing hospital readmissions, none of the studies conducted a detailed analysis of the readmissions among patients undergoing skin procedures. Furthermore, the results of previous studies provide limited information on the readmissions of these groups of patients (Golberg and Cho, 2004; Arnold et al., 2018). A retrospective cohort study of dermatology hospitalizations was conducted to evaluate the frequency and demographics of readmissions following skin disease (Arnold et al., 2018). The predictors for readmissions were the insurance type (Medicaid/Medicare), the economic conditions of the patients, and the number of chronic conditions faced by the patient. The size of the hospital and its location were also significant factors causing readmissions after a skin disease. Another recent study for patients with skin conditions analyzed the exact diagnosis and all-cause readmission. They found out that diseases contributing to the highest 30 days of readmission are different for both the same diagnosis and the use of readmission (Kassin et al., 2012). A potential limitation of these studies is the lack of detailed data on patient characteristics and a comprehensive data analysis approach that can provide meaningful insights.

Previous studies on readmissions have considered only one time interval, mostly 30 days (Dailey et al., 2013; ScienceDirect Topics, 2017; Arnold et al., 2018; Ofir and Padman, 2018; Zhang et al., 2019; Weiss et al., 2020). Some researchers (Dorajoo et al., 2017; Weiss et al., 2020) considered two time intervals in their research. Predictive modeling approaches such as logistic regression and various machine learning models were applied to analyze readmission rates in different research studies (Banks, 1998; Raykar and Saha, 2015; Arnold et al., 2018; Lee et al., 2018; Ofir and Padman, 2018; Zhang et al., 2019; Yu and Xie, 2020). Moreover, statistical models such as *t*-test and χ^2^ test were used to test hypotheses (Dorajoo et al., 2017). Furthermore, some features such as LOS, patient demographics, and hospital type were significant factors of readmission (Thomas et al., 1997; Lin et al., 2006; Kassin et al., 2012; Tsugawa et al., 2017). However, none of these studies provide a comprehensive analysis and comparison of different models and time intervals. Therefore, in the current study, we consider 30-day time intervals and seven machine learning models to find the most important predictors of the readmission of skin patients and the best model to predict the readmission. In addition, various factors such as patients' age, gender, claim type, line of business (LOB), month of admission, hospital key, and HCG subcategory are used to predict and analyze readmission.

## 3 Methodology

**Figure 1:**
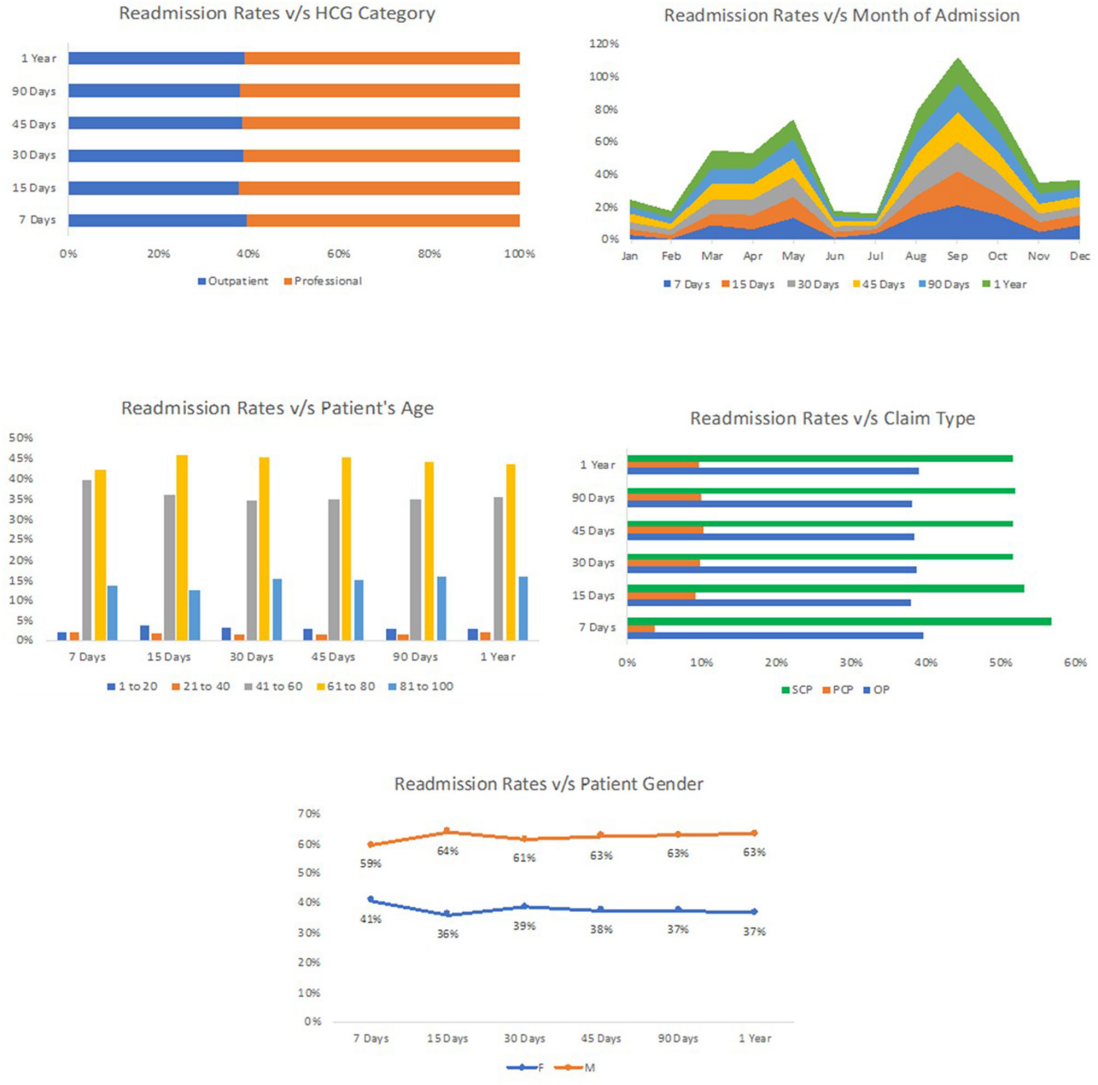
Readmission rates vs. all factors which were used for performing experiments.

The readmission rate in New York has been high, and the state has the 4th highest readmission rate in the United States for the FY 2020 (Empire Center for Public Policy, 2020). The data for this study were collected from the western part of New York, Rochester. This dataset consisted of information about all the admissions between July 2014 and June 2015. After cleaning the data, we found 22,388 records for the patients undergoing skin treatment within 1 year. Figure 1 shows the readmission rates versus all factors that were selected to perform experiments. The primary analysis showed the difference in the admission rate based on the treatment of patients. Therefore, the concentration groups were identified based on preliminary analysis, and data cleaning was performed to filter out the admissions occurring only at the place of service. The final reduced data displayed 2,272 records for the patients treated for skin-related disease at either an inpatient hospital, outpatient hospital, or under ambulatory surgery within 1 year. The three main places of service admission rates were ambulatory surgery 0.93%, inpatient hospital 0.83%, and outpatient hospital 8.93%. Moreover, the total number of patients with skin conditions readmitted for the same diagnosis was 136.

### 3.1 Descriptive statistics

Table 1 represents the descriptive statistics for the occurrence of readmission for different factors of interest.

**Table 1:** Readmission rate based on various factors.

**Variable**	**Level**	**Readmission Rate (%)**
Patient's gender	Men	7%
	Women	4%
Patient's age	0–20	7%
	21–40	13%
	41–60	6%
	61–80	4%
	81–100	6%
Claim type	OP	3%
	PCP	2%
	SCP	6%
LOB	Commercial	7%
	Medicare	5%
HCG subcategory	Outpatient	4%
	Professional	6%
Month of admission	January	5%
	February	7%
	March	8%
	April	8%
	May	7%
	June	6%
	July	1%
	August	3%
	September	5%
	October	4%
	November	5%
	December	6%
Time interval	30 Days	6%

OP, outpatient; PCP, primary care physician; SCP, state county plan.

**Figure 2:**
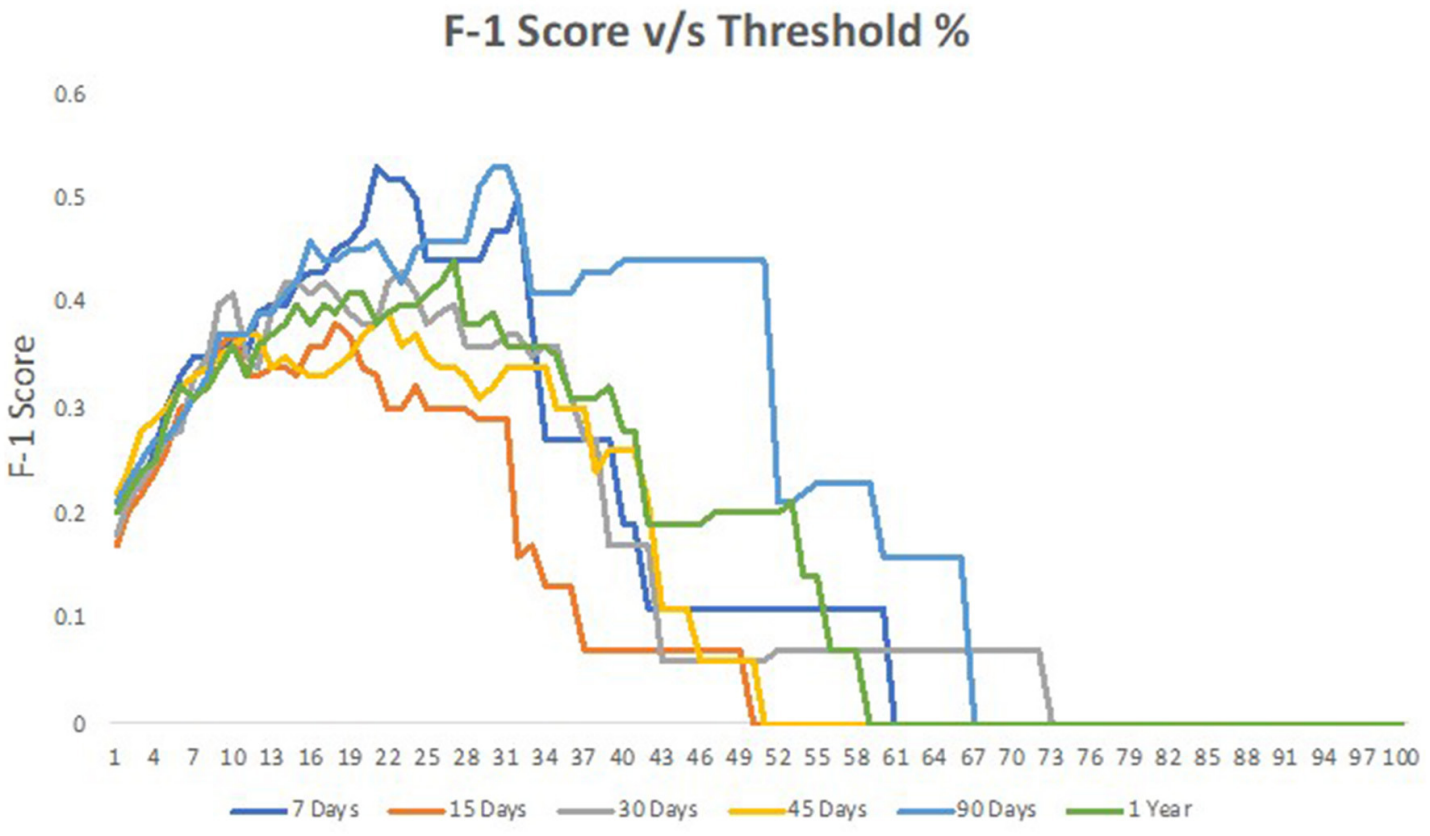
Performance of models across the percentage range.

Table 1 offers a detailed breakdown of readmission rates for skin patients across various factors. Starting with the gender of the patients, it is evident that men have a slightly higher likelihood of being readmitted, with a rate of 7%, compared with women at 4%. Age also plays a notable role in readmission. Patients between the ages of 21 and 40 exhibit the highest readmission rate at 13%. Interestingly, both the younger (0–20 years) and older age groups (81–100 years) share a similar readmission rate of 6%. Diving into claim types, “SCP” claims show the highest readmission rate of 6%, whereas “OP” and “PCP” have lower rates at 3 and 2%, respectively. When assessing the line of business (LOB), patients with commercial insurance face a higher readmission rate of 7%, in contrast to those on Medicare at 5%. The HCG subcategory, which likely refers to different healthcare groupings, indicates that professionals have a readmission rate of 6%, slightly higher than outpatients at 4%. Furthermore, monthly variations in readmissions are apparent, with March, April, and May seeing higher rates ranging from 7 to 8%. Interestingly, July has the lowest readmission rate at just 1%. Overall, when considering a 30-day time interval, the general readmission rate for skin patients stands at 6%. Figure 2 represents the performance of different models across the percentage range.

### 3.2 Machine learning

In medical research, machine learning techniques have gained prominence for forecasting various health outcomes such as mortality rates, patient readmissions, and duration of hospital stays. In this particular research, a dataset from upstate NY containing seven variables was used to apply seven machine learning approaches to estimate the probability of patient readmission within a 30-day timeframe. For model training, a 5-fold cross-validation was employed, using 80% of the data for training and the remaining 20% for validation. Given the data imbalance, the SMOTE technique was adopted (Brownlee, 2020), which creates artificial data points for the minority class, using the two closest data points for synthesis. This approach broadens the decision-making capacity of the minority class. Even though there is a potential for overfitting with oversampling, and it might demand more computational power, the SMOTE technique was found to be effective for this dataset, enhancing the predictive accuracy of the model.

## 4 Results

Table 2 provides a comparative analysis of various machine learning models and their performance metrics when predicting the risk of readmission for skin patients. The models are categorized into two groups: those that can calculate the importance of variables (LR, RF, and XG) and those that cannot (NB, KNN, ANN, and SVM). Performance metrics such as ACC, F-1, AUC, Precision, and Recall are used to evaluate the effectiveness of each model. For instance, RF and XG both demonstrate superior performance across all metrics, notably achieving an accuracy and F1 score of 0.85 and an AUC of 0.90 and 0.89, respectively. These high scores indicate that these models are particularly proficient at predicting readmissions among skin patients. In contrast, models such as NB and LR tend to have slightly lower scores, with NB's F1 score being 0.59 and LR's accuracy at 0.64. The table serves as a comprehensive guide for understanding the efficacy of each model in the context of predicting readmissions, which is crucial for medical professionals aiming to select the most appropriate predictive model for their needs.

**Table 2:** Importance measures of machine learning models.

	**Ability to calculate the importance of variables**			**Inability to calculate the importance of variables**			
	**LR**	**RF**	**XG**	**NB**	**KNN**	**ANN**	**SVM**
ACC	0.64	0.85	0.85	0.62	0.68	0.74	0.68
F-1	0.63	0.85	0.85	0.59	0.68	0.74	0.68
AUC	0.66	0.90	0.89	0.70	0.79	0.82	0.73
Precision	0.65	0.86	0.86	0.68	0.70	0.74	0.70
Recall	0.64	0.85	0.85	0.62	0.68	0.74	0.68

Three out of seven models, namely, XG, RF, and LR, were found to have the ability to identify the most relevant predictors of the risk of readmission in the study. Considering the results of Table 2, which indicate that the relationship between predictors is non-linear, and the dataset is large and complex, and since RF and XG show better performance, the variable importance of these models is more reliable.

Table 3 showcases the significance of different predictors in determining the readmission rates of skin patients as interpreted by three machine learning models, namely, RF, XG, and LR. Each value in the table represents the weight or importance assigned to that predictor by the respective model. For the RF model, the “Month of admission” predictor stands out with the highest weight of 0.355, indicating that the month in which a patient is admitted plays a significant role in predicting readmissions. The “Hospital Key” and “Age” also follow closely. In contrast, the XG model seems to prioritize the “LOB” and “Hospital Key”, with weights of 0.176 and 0.188, respectively.

**Table 3:** Most important predictors of readmission of skin patients.

	**RF**	**XG**	**LR**
LOB	0.062	0.176	−1.539
Hospital Key	0.230	0.188	0.484
HCG sub-category	0.022	0.152	0.397
Claim type	0.036	0.085	−0.014
Age	0.219	0.156	0.721
Gender	0.073	0.133	0.452
Month of admission	0.355	0.126	−0.007

## 5 Discussion and conclusion

The data provide a multifaceted insight into the factors influencing readmission rates for skin patients. A distinct disparity between male and female readmission rates suggests that sex-specific medical needs or social factors might influence readmission likelihood. Unsurprisingly, age plays a pivotal role; younger adults (21–40 years) exhibit a notably higher readmission rate, hinting at potential complications or post-operative care challenges in this age group. Interestingly, the consistency in readmission rates between the youngest and oldest age groups suggests overarching health challenges that might span both age extremes.

Among the evaluated machine learning models, RF and XG emerged as leaders in predictive performance. This superiority can be attributed to the inherent capabilities of these models. RF, which builds multiple decision trees and aggregates their results, is known for its high accuracy and ability to handle large data with higher dimensionality. XG, on the other hand, is an advanced implementation of gradient boosting which systematically refines its predictions over numerous iterations, often leading to improved results compared with other algorithms. Their ability to calculate and assign importance to variables makes them adept at identifying and leveraging nuanced patterns in complex datasets.

The prominence of “Month of admission,” “Age,” and “Hospital Key” as significant predictors in readmission rates is noteworthy. Variations in monthly readmission rates might reflect seasonal health challenges or hospital operation fluctuations, such as staffing. The significance of age, a universally accepted health determinant, underscores the potential medical challenges associated with skin conditions at different life stages. The importance of “Hospital Key” suggests that institutional factors, such as quality of care, available facilities, or post-operative procedures, play a crucial role in readmission likelihood.

In the intricate realm of medical readmissions, understanding and predicting patterns can lead to improved patient care and efficient resource allocation. While several variables contribute to readmission rates for skin patients, models such as RF and XG prove to be invaluable tools in deciphering these patterns. Their robust performance underscores their utility in medical predictions. The prominence of predictors such as age and hospital-specific factors reinforces the necessity for personalized patient care and the role institutions play in patient outcomes. As healthcare systems evolve, leveraging such insights can significantly enhance patient experiences and outcomes.

## Data availability statement

The data analyzed in this study is subject to the following licenses/restrictions: the data used in this paper is de-identified patient information that was provided by GRIPA to RIT. Requests to access these datasets should be directed to nafeie@rit.edu.

## Author contributions

JA worked on this research as part of his MSc. thesis and he was responsible for preparing the first draft and running the model and analysis. BB contributed to this paper by adding some discussion points, editing the manuscript, and helping to develop the models and report the analysis results. NA-F is the advisor of JA and BB, who advised them in preparing the manuscript, and reading and commenting on the methodology, results, and discussion. All authors contributed to the article and approved the submitted version.

## Footnotes

^1^Centers for Medicare and Medicaid Services Planned. Available online at: hscrc.maryland.gov/documents/HSCRC_Initiatives/readmissions/Version-2-1-Readmission-Planned-CMS-Readmission-Algorithm-Report-03-14-2013.pdf.

^2^ https://gis.cdc.gov/Cancer/USCS/#/AtAGlance/
